# Leisure-time physical activity and gastric cancer risk: A pooled study within the Stomach cancer Pooling (StoP) Project

**DOI:** 10.1371/journal.pone.0286958

**Published:** 2023-07-12

**Authors:** Marco Mariani, Roberta Pastorino, Denise Pires Marafon, Ken C. Johnson, Jinfu Hu, Antonio Jose Molina de la Torre, Guillermo Fernández-Tardón, David Zaridze, Dmitry Maximovich, Eva Negri, Carlo La Vecchia, Zuo-Feng Zhang, Robert C. Kurtz, Claudio Pelucchi, Matteo Rota, Stefania Boccia

**Affiliations:** 1 Section of Hygiene, University Department of Life Sciences and Public Health, Università Cattolica del Sacro Cuore, Rome, Italy; 2 Department of Woman and Child Health and Public Health, Fondazione Policlinico Universitario A. Gemelli IRCCS, Rome, Italy; 3 School of Epidemiology and Public Health, Department of Medicine University of Ottawa, Ottawa, Ontario, Canada; 4 Harbin Medical University, Harbin, China; 5 Biomedicine Institute (IBIOMED), University of León, León, Spain; 6 Consortium for Biomedical Research in Epidemiology and Public Health (CIBERESP), Madrid, Spain; 7 Health Research Institute of Asturias, ISPA and IUOPA, University of Oviedo, Oviedo, Spain; 8 Department of Clinical Epidemiology, N.N.Blokhin National Medical Research Center for Oncology, Moscow, Russia; 9 Department of Medical and Surgical Sciences, University of Bologna, Bologna, Italy; 10 Department of Clinical Sciences and Community Health, University of Milan, Milan, Italy; 11 Department of Epidemiology, UCLA Fielding School of Public Health and Jonsson Comprehensive Cancer Center, Los Angeles, CA, United States of America; 12 Department of Medicine, Memorial Sloan Kettering Cancer Centre, New York, NY, United States of America; 13 Department of Molecular and Translational Medicine, Università degli Studi di Brescia, Brescia, Italy; University of Pavia, ITALY

## Abstract

**Background:**

Although physical activity (PA) has been recognized as a favourable factor in the prevention of various diseases, including certain forms of cancer, the relationship between PA and gastric cancer (GC) is not yet fully understood. This study aims to provide data from a pooled analysis of case-control studies within the Stomach cancer Pooling (StoP) Project to estimate the association between leisure-time PA and the occurrence of GC.

**Methods:**

Six case-control studies from StoP project collected data on leisure-time PA, for a total of 2,343 cases and 8,614 controls. Subjects were classified into three leisure-time PA categories, either none/low, intermediate or high, based on study-specific tertiles. We used a two-stage approach. Firstly, we applied multivariable logistic regression models to obtain study-specific odds ratios (ORs) and corresponding 95% confidence intervals (CIs) then, we used a random-effect models to obtain pooled effect estimates. We performed stratified analyses according to demographic, lifestyle and clinical covariates.

**Results:**

The meta-analysis showed ORs of GC with no significant differences between intermediate vs low and high vs low PA level (OR 1.05 [95%CI 0.76–1.45]; OR 1.23 [95%CI 0.78–1.94], respectively). GC risk estimates did not strongly differ across strata of selected covariates except for age ≤ 55 years old (high vs low level: OR 0.72 [95%CI 0.55–0.94]) and for control population-based studies (high vs low level: OR 0.79 [95%CI 0.68–0.93]).

**Conclusions:**

No association was found between leisure time PA and GC, apart from a slight suggestion of decreased risk below age 55 and in control population-based studies. These results may reflect specific characteristics of GC at a younger age, or the presence of a cohort effect mediating and interacting with socioeconomic determinants of GC The different distribution of PA levels among hospitalized controls could have led to an underestimated effect of PA on GC risk.

## Introduction

Gastric cancer (GC) is one of the most common cancers worldwide, particularly in low income countries [1]. Over the last few decades, a decline of incidence rates was observed, mainly because of the recognition and control of *H*. *Pylori* infection, plus other dietary and environmental factors [2, 3]. However, the trend in incidence of cardia GC remained stable or increased in the Western countries [4]. Physical activity represents one of the most important modifiable determinants of all-cause mortality and non-communicable diseases [5]. In particular, recreational PA largely incorporates various activities undertaken during leisure time and represents a potentially modifiable component of energy expenditure [6]. It is reported that more than 25% of the adult population worldwide (1.4 billion adults, i.e. 1 in 3 women and 1 in 4 men) do not practice enough PA, with a particularly high level of inactivity across the high-income cohort [7]. PA is also a predictor of overall mortality with an estimated 4 to 5 million deaths per year that could be avoided if the global population were more physically active [8]. PA has been considered in relation to GC, with somewhat inconsistent results [9]. Among plausible favorable pathways, there are molecular pathways that contribute to genome instability in key growth regulatory genes propelled by endogenous and exogenous factors, such as: decreased systemic inflammation, hyperinsulinemia and insulin resistance, insulin-like growth factor (IGF-I), steroid hormones, dysregulated level of leptin, obesity-related cytokines, adiponectin and, diversity in the composition of the gut microbiota [10–14]. There is limited evidence, however, between PA and GC [2] and it is important to assess if having an active lifestyle could reduce the risk of developing such form of cancer. In fact, when studying the association between PA and GC, most studies did not provide adequate data for properly adjusting for potential confounders such as smoking, socioeconomic status and dietary habits. The objective of this study was to explore the association between leisure-time PA and GC risk through a pooled analysis of case-control studies within the International “Stomach cancer Pooling Project” (StoP) [15], and to further assess this relationship in strata of selected covariates.

## Material and methods

### Studies and participants

Overall, ten out of over 30 studies included in the latest release (number 2.1) of the StoP dataset collected data on leisure-time PA [16–25]. However, four studies [22–25] were not considered due to missing data (>30%) [26] or their qualitative nature. Six studies were included in the pooled analysis: Italy 1 [20], Italy 2 [21], Canada [19], Russia [18], USA [17], and Spain [16]. All the studies participating in the StoP consortium [15] were conducted in accordance with applicable laws, regulations and guidelines for protection of human subjects, and the StoP Project received ethical approval from the University of Milan Review Board (reference no. 19/15 of 01/04/2015).

### Exposure assessment and data standardization

The questionnaires usually included demographic and lifestyle data on PA, cigarette smoking, alcohol use, dietary habits, and family history of cancer. Additional data were obtained from cancer registries or hospital medical records. All data were collected and standardized according to a pre-specified format at the data-pooling center.

The main characteristics of PA variables are outlined in **Table 1**. Studies reported [16–21] the duration (number of hours) of leisure-time PA over a certain interval of time (a week period). In particular, Italian and US centers [17, 20, 21] had pre-specified criteria with cut-off points in their questionnaires, while Canada, Russia and Spain [16, 18, 19] reported continuous values. Study subjects were assigned to one of three PA categories, either none/low, intermediate, or high. These cut-offs were selected using the WHO and US 2018 guidelines threshold for the upper limit of 300 minutes (5 hours) and 120 minutes, instead of the recommended 150 minutes threshold [27, 28], for the lower limit (due to unavailability of data). None/low level of exposure were combined due to the nature of individual study questionnaires and were considered as a reference category.

**Table 1:** Study specific definitions for physical activity.

Study center	Study-specific definition	Study-specific tertiles
Italy 1 (Negri)	Sport, leisure, activities, bicycle rides at various ages (12,15–19,30–39,50–59).	None/Low: <2 hours per week
		Intermediate: 2–4 hours per week
		High: ≥5 hours per week
Italy 2 (Boccia)	Walking, cycling, taking care of the garden or house, gym and other athletic activities	None/Low: <2 hours per week
		Intermediate: 2–4 hours per week
		High: ≥5 hours per week
Canada	Number of hours per week spent doing both moderate and strenuous activities (walking, jogging, gardening, home exercises, golf, racquet sports, bowling, swimming, skiing or skating, bicycling, social dancing and other) averaged over seasons and related to 2 years before the interview.	None/Low: <2 hours per week
		Intermediate: 2–4 hours per week
		High: ≥5 hours per week
Russia	Walking, sport and gardening activities (hours per week) during summer/winter seasons and referring to 1 year preceding the disease (cases and hospital controls) and 1 year prior to the interview for visitor controls.	None/Low: <2 hours per week
		Intermediate: 2–4 hours per week
		High: ≥5 hours per week
USA	Active sports, physical exercise, jogging-running, swimming/long walks, gardening/fishing/hunting, other activities	None/Low: <2 hours per week
		Intermediate: 2–4 hours per week
		High: ≥5 hours per week

### Data analysis

The relationship between PA and GC was evaluated using a two-stage approach [29]. Firstly, multivariable logistic regression models were applied to obtain study-specific odds ratios (ORs) and the corresponding 95% Confidence Intervals (CIs). The following covariates were included in the logistic regression models: sex, age, smoking, alcohol consumption, body mass index (BMI), social class, occupational PA, cancer history, and dietary habits. For covariates with up to 10% missing values (BMI, social class, smoking status, alcohol consumption, and vegetable and fruit intake) we performed multiple imputation including the same set of covariates in the analysis model.

In the second phase, a random-effect model was applied in order to estimate summary (pooled) effect measures. Heterogeneity across studies was assessed with the Q and *I*^*2*^ statistics measures [30]. In order to investigate the effects of leisure-time PA across strata of selected covariates, we performed stratified analyses according to: sex, age (≤55, 56–65, >65), BMI (underweight/normal weight, overweight, obese), socioeconomical status (study-specific low, intermediate, high), cigarette smoking status (never, former, current smoker), alcohol drinking status (study specific levels: never, low level of consumption, intermediate and high level of consumption), vegetables and fruit intake (study-specific low, intermediate, high), occupational PA (study-specific low, intermediate, high), cancer history among first degree relatives (yes, no), GC anatomical site (cardia, non-cardia), GC histological type (intestinal, diffuse, undifferentiated), type of controls (population, hospital) and *H*. *pylori* status (positive, negative). Since only some studies had information on *H*. *pylori* infection, we also carried out calculations using the random-effects model and performed sensitivity analyses including only those individuals that were *H*. *pylori* positive. Heterogeneity tests were performed across all the strata estimates. Statistical analyses were carried out using STATA software, version 16.

## Results

The main characteristics of the 2,343 cases and 8,614 controls included in the present analysis are reported in **Table 2**.

**Table 2:** Distribution of 2,343 cases of gastric cancer and 8,614 controls according to selected covariates.

	Cases	Controls	chi^2^ test
	N = 2,343	N = 8,614	
	n (%)	n (%)	p value
**Study Center**			-
Italy 1	221 (9.4)	525 (6.1)	
Italy 2	160 (6.8)	444 (5.2)	
Canada	1,182 (50.5)	5,039 (58.5)	
Russia	450 (19.2)	611 (7.1)	
USA	132 (5.6)	132 (1.5)	
Spain	198 (8.5)	1,863 (21.6)	
**Physical activity** ^a^			0.71
Low	1,272 (54.3)	4,595 (53.3)	
Intermediate	525 (22.4)	1,962 (22.8)	
High	546 (23.3)	2,057 (23.9)	
**Sex**			<0.0001
Male	1,496 (63.9)	4,495 (52.2)	
Female	847 (36.1)	4,119 (47.8)	
**Age at diagnosis or interview**			<0.0001
≤ 55 yrs	530 (22.6)	3,001 (34.8)	
56–65 yrs	689 (29.4)	2,270 (26.4)	
> 65 yrs	1,124 (48.0)	3,343 (38.8)	
**BMI Classification** ^b^			0.39
Under or normal weight	1,022 (47.4)	3,703 (45.9)	
Overweight	803 (37.2)	3,129 (38.7)	
Obese	332 (15.4)	1,243 (15.4)	
*Missing*	*186 (7*.*9)*	*539 (6*.*3)*	
**Socioeconomical status**			<0.0001
Low	886 (39.7)	2,933 (35.6)	
Intermediate	913 (41.0)	3,012 (36.6)	
High	430 (19.3)	2,290 (27.8)	
*Missing*	*114 (4*.*9)*	*379 (4*.*4)*	
**Cigarette smoking status**			0.003
Never	846 (39.5)	3,608 (43.1)	
Former	866 (40.5)	3,065 (36.7)	
Current	428 (20.0)	1,690 (20.2)	
*Missing*	*203 (8*.*7)*	*251 (2*.*9)*	
**Alcohol drinking status**			<0.0001
Never	519 (24.8)	2,098 (26.9)	
Low	683 (32.6)	3,377 (43.3)	
Intermediate	542 (25.9)	1,716 (22.0)	
High	349 (16.7)	603 (7.8)	
*Missing*	*250 (10*.*7)*	*820 (9*.*5)*	
**Vegetables and fruit intake**			<0.0001
Low	627 (27.4)	2,693 (32.0)	
Intermediate	754 (33.0)	2,739 (32.6)	
High	905 (39.6)	2,974 (35.4)	
*Missing*	*57 (2*.*4)*	*208 (2*.*4)*	
**Occupational PA** ^c^			<0.0001
Low	297 (35.8)	1,083 (38.4)	
Intermediate	333 (40.1)	1,284 (45.6)	
High	200 (24.1)	450 (16.0)	
*Missing*	*1*,*513 (64*.*6)*	*5*,*797 (67*.*3)*	
**Family history of GC** ^d^			<0.0001
Yes	156 (13.8)	243 (6.9)	
No	978 (86.2)	3,274 (93.1)	
*Missing*	*1*,*209 (51*.*6)*	*5*,*097 (59*.*2)*	
**GC anatomical site**			-
Cardia	560 (35.3)	-	
Non-Cardia	1,026 (64.7)	-	
*Missing*	*757 (32*.*3)*	*-*	
**GC histological type**			-
Intestinal	409 (19.3)	-	
Diffuse	325 (15.3)	-	
Undifferentiated	1,388 (65.4)	-	
*Missing*	*221 (9*.*4)*	*-*	
**Type of control**			-
Hospital	-	1,712 (19.9)	
Population	-	6,902 (80.1)	
***H*. *pylori* status** ^e^			<0.0001
Positive	381 (60.6)	1,253 (77.5)	
Negative	248 (39.4)	363 (22.5)	
*Missing*	*1*,*714 (73*.*2)*	*6*,*998 (81*.*2)*	

^a^ details regarding the classification of PA variable available in S1 Table

^b^ underweight, normal or healthy weight (below 24.9), overweight (25.0–29.9) and obese (30.0 and above)

^c^ available for three centers (Italy 1, Russia and Spain)

^d^ information for cancer history was not available for Canadian center

^e^ available for three centers (Italy 2, Russia and Spain).

The majority of study subjects both among cases and controls showed low levels of PA (54.3% among cases and 53.3% among controls). On average, cases were older than controls (62.9 vs 58.9 years of age) and of lower social classes (39.7% in cases vs 35.6% in controls). The proportion of current smokers was similar across cases (20.0%) and controls (20.2%), while cases were more than twice as likely as to consume high levels of alcoholic beverages (16.7% vs 7.8%, respectively). The vast majority were non-cardia site (64.7%). Regarding information on *H*. *pylori* status, the data were only available for three study centers (Italy 2, Russia and Spain, in particular, in the Italy 2 study, information on *H*. *pylori* was available only for cases).

The ORs and 95% CI for GC according to leisure PA levels are presented in **Table 3**.

**Table 3:** Pooled and adjusted odds ratios (aORs) and 95% confidence intervals (CIs) for gastric cancer according to leisure physical activity levels overall (reference: Low level of physical activity) and by strata of selected covariates (sex, age, socioeconomic status, BMI, cigarette smoking, alcohol drinking, vegetables and fruit intake, occupational PA, cancer anatomical site, cancer histotype, type of controls, *H*. *pylori* infection, and GC family history).

	Intermediate vs low				High vs low			
	Study (n)	aOR(95%CI)^a^	I^2^(%)	p^b^	Study (n)	aOR(95%CI) ^a^	I^2^(%)	p^b^
**Overall**	6	1.05 (0.76–1.45)	76.0	0.001	6	1.23 (0.78–1.94)	87.9	<0.0001
**Sex**								
Male	6	1.15 (0.78–1.69)	69.5	0.006	6	1.30 (0.81–2.09)	80.1	<0.0001
Female	6	0.85 (0.66–1.10)	16.5	0.31	6	1.15 (0.69–1.94)	72.9	0.002
**Age at diagnosis or interview**								
≤ 55 yrs	6	0.85 (0.65–1.11)	0.0	0.46	6	**0.72 (0.55–0.94)**	0.0	0.66
56–65 yrs	6	0.91 (0.66–1.27)	21.6	0.27	6	1.39 (0.81–2.38)	63.9	0.017
> 65 yrs	6	1.01 (0.61–1.66)	73.6	0.002	6	1.44 (0.65–3.20)	90.1	<0.0001
**BMI Classification**								
Under or normal weight	6	0.90 (0.62–1.32)	59.6	0.030	6	1.13 (0.68–1.90)	78.7	<0.0001
Overweight	6	1.19 (0.78–1.80)	62.7	0.020	6	1.18 (0.70–1.99)	72.8	0.002
Obese	5/6*	0.86 (0.59–1.26)	0.0	0.53	5/6	1.42 (0.89–2.28)	21.1	0.28
**Socioeconomical status**								
Low	6	1.08 (0.70–1.69)	55.5	0.047	6	1.47 (0.70–3.10)	86.1	<0.0001
Intermediate	6	1.00 (0.78–1.29)	15.2	0.32	6	0.98 (0.77–1.25)	12.0	0.34
High	6	0.76 (0.47–1.22)	35.4	0.17	6	0.80 (0.60–1.06)	0.0	0.55
**Cigarette smoking status**								
Never	6	1.01 (0.70–1.44)	55.6	0.046	6	1.07 (0.71–1.60)	63.0	0.019
Former	6	1.06 (0.70–1.60)	48.3	0.085	6	1.34 (0.74–2.40)	71.7	0.003
Current	6	0.83 (0.54–1.28)	31.5	0.20	6	1.26 (0.70–2.28)	62.6	0.020
**Alcohol drinking status**								
Never	6	0.88 (0.65–1.18)	3.7	0.39	6	1.45 (0.80–2.64)	68.8	0.007
Low	4/6*	1.03 (0.64–1.65)	63.4	0.042	4/6*	0.83 (0.58–1.20)	29.6	0.24
Intermediate	6	0.77 (0.58–1.02)	0.0	0.57	6	1.08 (0.66–1.77)	57.9	0.036
High	4/6*	0.95 (0.58–1.57)	18.3	0.30	4/6*	0.85 (0.58–1.25)	0.0	0.43
**Vegetables and fruit intake**								
Low	5/6*	0.94 (0.72–1.24)	0.0	0.55	5/6*	0.85 (0.65–1.12)	0.0	0.45
Intermediate	5/6*	1.01 (0.57–1.80)	76.1	0.002	5/6*	0.98 (0.77–1.24)	0.0	0.63
High	6	0.82 (0.66–1.02)	0.0	0.45	6	1.07 (0.66–1.74)	74.3	0.002
**Occupational PA** ^c^								
Low	3	1.28 (0.68–2.41)	65.1	0.057	3	1.36 (0.87–2.13)	7.3	0.34
Intermediate	3	0.99 (0.69–1.42)	0.0	0.99	3	0.87 (0.59–1.29)	0.0	0.69
High	3	0.99 (0.57–1.70)	0.0	0.95	3	0.86 (0.51–1.47)	0.0	0.77
**Family history of GC** ^d^								
Yes	3/5*	1.18 (0.59–2.35)	0.0	0.44	3/5*	0.87 (0.31–2.42)	33.3	0.22
No	5	1.24 (0.92–1.69)	46.5	0.11	5	1.39 (0.84–2.29)	78.3	0.001
**GC anatomical site**								
Cardia	6	1.07 (0.71–1.60)	40.4	0.14	6	1.43 (0.94–2.18)	44.4	0.11
Non-cardia	6	0.89 (0.61–1.31)	69.9	0.005	6	0.99 (0.58–1.69)	83.9	<0.0001
**GC histological type**								
Intestinal	6	**1.38 (1.03–1.84)**	0.0	0.91	6	**1.81 (1.17–2.78)**	50.8	0.071
Diffuse	6	1.02 (0.74–1.40)	2.7	0.40	6	1.34 (0.92–1.94)	21.8	0.27
Undifferentiated	5/6*	1.00 (0.62–1.61)	65.4	0.021	6	0.89 (0.65–1.23)	27.2	0.23
**Type of controls**								
Population	2/2	0.76 (0.55–1.05)	60.8	0.11	2/2	**0.79 (0.68–0.93)**	0.0	0.37
Hospital	4/4	1.26 (0.98–1.63)	14.6	0.32	4/4	1.53 (0.79–2.99)	84.9	<0.0001
***H*. *pylori* status** ^e^								
Positive	2/3*	1.09 (0.75–1.57)	0.0	0.56	2/3*	1.07 (0.73–1.58)	0.0	0.70
Negative	2/3*	0.94 (0.55–1.59)	0.0	0.85	2/3*	1.68 (0.96–2.95)	0.0	0.54

^a^ Pooled ORs were computed using random-effects models using the non-low level of physical activity category as reference

^b^ p for heterogeneity

^c^ available for three centers (Italy 1, Spain and Russia)

^d^ Information for family history of GC was not available for Canadian center

^e^ available for both cases and controls in two centers (Russia and Spain)

*only computable for the number of studies indicated in the numerator respect to the denominator (total number).

**Fig 1:**
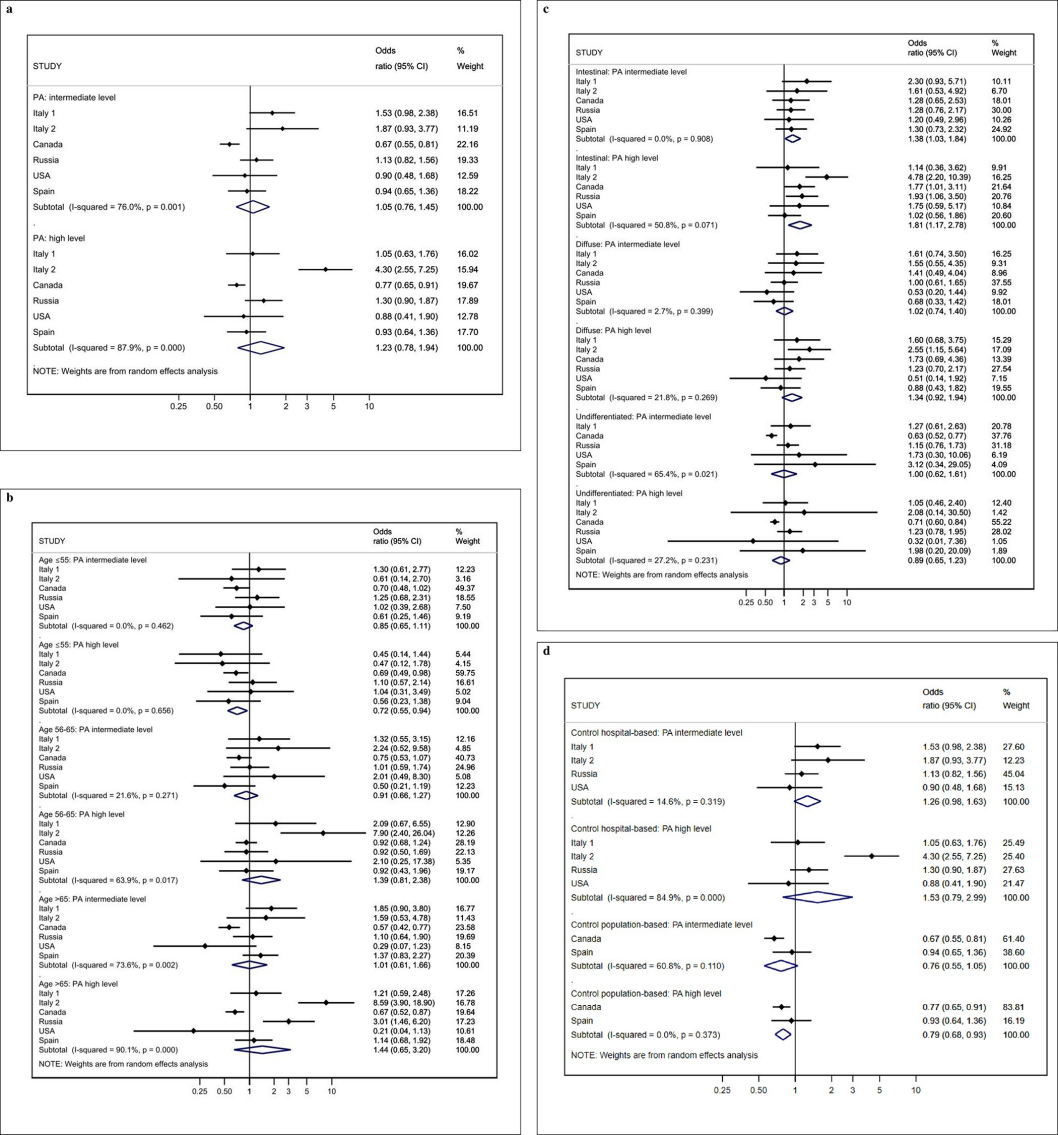
Forest plot representing the overall association of gastric cancer and physical activity (intermediate or high level of physical activity respect to low level of physical activity) (a) and by categories of age (b), histological type (c) and type of control (d).

Overall, we did not find significant differences across PA levels (**Table 3; Fig 1A)** with the exception for age strata, the histological type of GC and the type of control. Among subjects ≤ 55 years, high levels of PA showed an OR of 0.72 (95%CI 0.55–0.94) (**Fig 1B**). For the intestinal type of GC we obtained an OR of 1.38 (95%CI 1.03–1.84) and 1.81 (95%CI 1.17–2.78) for the intermediate and high level of PA, respectively, compared to the low levels (**Fig 1C**). For control population-based studies, high levels of PA showed an OR of 0.79 (0.68–0.93) (**Fig 1D**).

The analysis restricted to *H*. *pylori* positive individuals (Russian and Spanish studies) reported no differences between low levels of PA and intermediate or high levels over all strata with the only exception for family history of GC. Subjects with family history of GC performing high levels of PA, respect to low level of PA, had an OR of 0.18 (95%CI 0.03–0.96).

## Discussion

We did not find an association between leisure-time PA and GC risk with no substantial differences in the risk estimates according to occupational PA strata. Our results do not parallel the findings from a meta-analyses [31] that included 10 cohort and 12 case-control studies, whose results showed a 19% reduced risk for GC among persons in the highest category of any PA compared to the lowest [31]. However, when the authors restricted the analysis to data on recreational PA and included nine case-control studies (3,045 cases and 21,128 controls) and seven cohort studies (4,814 cases), the meta-analysis reported non-significant effect estimates (pooled OR for case-control studies: OR = 0.86 [95% CI (0.69–1.07)]; pooled risk ratio for cohort studies RR = 0.92 [95% CI (0.74–1.15)]. Significant protective effects, however, were reported from two cohort studies including non-cardia GC cases (pooled RR = 0.62; 95% CI: 0.52–0.75) [31]. Additional pooled analyses were reported by another study that analyzed 12 US and EU cohorts that showed non-significant association among 1,428 non-cardia GC cases (hazard ratio (HR) = 0.92, 95% CI: 0.73, 1.15) when comparing high versus low levels of leisure-time PA [32]. Furthermore, a recent meta-analysis [33] showed that GC risk is lower among people with high PA level compared to those with low level of PA with a relative risk of 0.83, 95%CI (0.76–0.91) [33]. Our results show no substantial differences in the risk estimates according to occupational PA strata. As to occupational PA, Our findings are somewhat expected, since previous research reported no evidence of the effect of occupational PA on GC risk [34, 35]. The European Prospective Investigation into Cancer and Nutrition (EPIC) cohort observed no increase in risk of GC among sedentary occupations in comparison to manual or standing occupations [36]. Individuals of lower socio-economic positions and employed in manual occupations tend to engage in less leisure-time PA [37] and there can be occupation-specific chemical and environmental exposures associated to higher risk of GC (wood dust, aromatic amines, pesticides and herbicides, coal derivatives, chromium etc.) [38]. Thus, our estimates may be influenced by under-adjustment for social class indicators. However, a relationship between job position and health outcomes is far more complex and prone to influence of various factors including socioeconomic indicators such as education and wealth [39]. The EPIC data reported a reduction in GC risk among populations of higher educational level [40]. In fact, low education is linked to an increased risk of GC and other main lifestyle risk factors for GC (tobacco, alcohol, fruit and vegetable consumption, processed meat consumption and salt intake) mediate only about 10% of the difference in GC risk between highly and less educated individuals. The *H*. *pylori* may be involved in this difference as it is also more frequently reported among low people of low socioeconomic status [41, 42]. In fact, as reported in a nested case-control study from the EPIC cohort, these effects were largely attenuated after adjusting for *H*. *pylori* [40].

A limitation is represented by recreational PA data collected by study-specific questionnaires with different definitions and classifications. To have comparable variables, we defined three levels of PA intensity based on defined thresholds and wherever possible using the standard ones [42]. This might have led to some limitation in accounting for the type of PA in terms of energy expenditure and its adjustment according to the type of body mass, i.e. percentage of muscle or body fat, since people with higher percentages of fat compared to people with lower percentages of fat composition show different energy expenditure. In addition, data on PA was collected at different time points prior to GC diagnosis, i.e. from one year up to 5 years and this might have contributed to non-differential misclassification of PA exposure.

Moreover, the stratified analysis regarding the intestinal type of GC showed an opposing trend respect to both the overall analysis, on association of GC and PA, and the analysis stratified by categories of age which may be due to an under-adjustment. Furthermore, adjustments for social class position in our statistical models are of particular importance in the present research and a role of under-adjustment is possible.

Lastly, we have three studies with hospital-based controls. These controls may have a different distribution of PA levels because individuals who are hospitalized may be more likely to have underlying health conditions that affect their ability to engage in PA. This may have underestimated the effect of PA on GC risk.

## Conclusions

We did not find an association between PA levels and GC risk, apart from some suggestion of decreased risk below age 55. This may reflect specific characteristics of GC at a younger age, or the presence of a cohort effect mediating and interacting with socioeconomic determinants of GC. A decreased risk also emerged when we analyzed control population-based studies separately, possibly due to the distribution of PA levels in the hospitalized controls leading to an underestimation of the effect of PA on GC risk.

## Supporting information

S1 Table Study specific definitions for PA. Stomach cancer pooling (StoP) Project consortium.
(DOCX)
