# Supplementary material for: Leisure-time physical activity and gastric cancer risk: A pooled study within the Stomach cancer Pooling (StoP) Project
Source: PLoS One. 2023 Jul 12;18(7):e0286958. doi: 10.1371/journal.pone.0286958 (PMC10337950; doi:10.1371/journal.pone.0286958)
Supplement: S1 Table — Stomach cancer pooling (StoP) Project consortium. (DOCX) [file pone.0286958.s001.docx]

**Table S1.** Study specific definitions for PA. Stomach cancer pooling (StoP) Project consortium.

| **Study center** | **Study-specific definition** | **Study-specific PA levels** |
| --- | --- | --- |
| Italy 1 (Negri) | Sport, leisure, activities, bicycle rides at various ages (12,15-19,30-39,50-59). | None/Low: <2 hours per week Intermediate: 2-4 hours per week High: ≥5 hours per week |
| Italy 2 (Boccia) | Walking, cycling, taking care of the garden or house, gym and other athletic activities | None/Low: <2 hours per week Intermediate: 2-4 hours per week High: ≥5 hours per week |
| Canada | Number of hours per week spent doing both moderate and strenuous activities (walking, jogging, gardening, home exercises, golf, racquet sports, bowling, swimming, skiing or skating, bicycling, social dancing and other) averaged over seasons and related to 2 years before the interview. | None/Low: <2 hours per week Intermediate: 2-4 hours per week High: ≥5 hours per week |
| Russia | Walking, sport and gardening activities (hours per week) during summer/winter seasons and referring to 1 year preceding the disease (cases and hospital controls) and 1 year prior to the interview for visitor controls. | None/Low: <2 hours per week Intermediate: 2-4 hours per week High: ≥5 hours per week |
| USA | Active sports, physical exercise, jogging-running, swimming/long walks, gardening/fishing/hunting, other activities | None/Low: <2 hours per week Intermediate: 2-4 hours per week High: ≥5 hours per week |
| Spain | PA defined as activities that took place outside working hours, including walking, doing some sport, going to the gym, etc. in the last 5 years and for a period of at least 6 months (excluding 1 year prior to diagnosis) | None/Low: ≤2.9 hours per week  Intermediate: 3.0 - 5.6 hours per week  High: ≥5.7 hours per week |
